# Prevalence of Ca Blood Type and Alloantibodies in a Population of Horses from Italy

**DOI:** 10.3390/ani10071179

**Published:** 2020-07-13

**Authors:** Daniela Proverbio, Roberta Perego, Luciana Baggiani, Francesco Ferrucci, Enrica Zucca, Federico Nobile, Eva Spada

**Affiliations:** 1 Veterinary Transfusion Research Laboratory (REVLab), Department of Veterinary Medicine (DIMEVET), University of Milan, via dell’Università 6, 26900 Lodi, Italy; luciana.baggiani@unimi.it (L.B.); eva.spada@unimi.it (E.S.); 2 Department of Health, Animal Science and Food Safety (VESPA), University of Milan, via dell’Università 6, 26900 Lodi, Italy; Francesco.ferrucci@unimi.it (F.F.); enrica.zucca@unimi.it (E.Z.); 3 Veterinary pratictioner, Via Budrio 44, 20153 Milan, Italy; federiconobile@gmail.com

**Keywords:** equine, blood group, breed prevalence, blood type, alloantibodies, Italy, neonatal isoerythrolysis

## Abstract

**Simple Summary:**

Indications for whole blood transfusion in equine critical care include severe anemia from surgical blood loss or acute hemorrhage, hemolysis and neonatal isoerythrolysis. In horses, as in other animals, transfusions are associated with a number of inherent risks such as transfusion reactions. Pretransfusion screening and blood typing are indicated to minimize the risk of incompatible red cell transfusions. Equine blood types include seven systems, namely A, C, D, K, P, Q, and U. The major RBC antigens that warrant identification before packed RBC or whole blood transfusions in horses are Ca and Aa. The frequencies of blood groups can vary from one population to another and from one breed to another. In some situations where testing compatibility is not possible, such as in rural practice, the knowledge of the breed blood type frequencies may help selecting the best donor candidate. The aims of this study were to: estimate the prevalence of Ca blood type in horses from northern Italy; **estimate** the association between Ca blood type sex and breed of horse; estimate the prevalence of anti-Ca alloantibodies in Ca− horses. The prevalence of the Ca+ blood type was 79.1%. No significant association was found between blood type Ca and sex. The total number of Ca− samples with detectable anti-Ca alloantibodies was 7/23 (30.4%).

**Abstract:**

A knowledge of the blood groups and alloantibodies present is essential for the safe transfusion of blood products in horses. Pre-transfusion screening and blood typing minimizes the risk of incompatible RBC transfusions and prevents immunization of the recipient against incompatible RBC antigens. The frequencies of blood groups can vary among different breeds. Knowledge of a breed’s blood group prevalence can be very useful for identifying the best blood donors during transfusion in clinical practice. The aims of this study were to estimate the prevalence of the Ca blood type in horses from Italy using a monoclonal immunocromatographic method and to estimate the prevalence of anti-Ca alloantibodies in Ca− horses using agglutination on gel technique. Ca blood type was determined on 110 whole blood samples. The prevalence of the Ca+ blood type was 79.1%. This study also provides data about the prevalence of Ca+ blood group in Italian Saddle Horses (77,3%) and Dutch Warmblood (58,3%). No significant association was found between Ca blood type and sex with 79.5% and 78.8% of females and males testing Ca+, respectively. The total number of Ca− samples with detectable anti-Ca alloantibodies was 7/23 (30.4%).

## 1. Introduction

Blood group analysis plays a pivotal role in human and animal health. Blood groups are the result of inherited species-specific antigens on the surface of the red blood cells (RBC). These antigens play an important role in inducing immune-mediated reactions and can cause complications following the transfusion of blood of different blood groups. Furthermore, natural alloantibodies may occur in plasma and can react against other blood groups without any prior exposure to the erythrocyte antigens. Equine blood types include 7 systems recognized by the International Society for Animal Genetics, A, C, D, K, P, Q, U that correspond to a particular gene for which two or more alleles exist. Each system contains from 1 to 15 factors, composed of proteins or carbohydrates [1], representing antigenic sites on the red blood cell surface. Blood groups are named with an uppercase letter to denote the system and a lowercase letter to designate the factor (a, b, c). Since more than one system can be co-expressed on the surface of equine red blood cells, this complex mechanism gives rise to more than 400,000 possible equine blood types [2] and makes it very unlikely that two unrelated horses (even if from the same breed) will share identical blood groups [1].

Horses can have two types of antibodies against blood group antigens that they lack; naturally occurring antibodies (present since birth and without the need for prior sensitization by transfusion or pregnancy) and, acquired antibodies (that develop following blood transfusion with incompatible whole blood or products containing erythrocytes or their antigens) [3]. Nevertheless, approximately 90% of horses do not possess natural alloantibodies against erythrocyte antigens, and not all horses develop antibodies after a single incompatible transfusion [4]. The most common naturally occurring antibodies are against Aa and Ca blood groups. Therefore, horses that lack the Aa antigen on the RBC frequently have anti-Aa antibodies, and horses that lack the Ca antigen on the RBC typically have anti-Ca antibodies [3]. 

Red blood cell antibodies are also involved in an uncommon foal disease, neonatal isoerythrolysis (NI). Neonatal isoerythrolysis can develop only when a mare is bred to a stallion with blood group for which she is negative, and the foal inherits this blood group. The mare may be sensitized by exposure to the foal’s blood during foaling or following a previous blood transfusion or previous pregnancy and thus produce antibodies against the foal’s RBC. When the foal ingests colostrum, it passively absorbs the antibodies against its RBC. Once in the blood, alloantibodies attach to the neonatal erythrocytes stimulating direct cell lysis or agglutination, and a hemolytic crisis occurs [5]. 

Whole blood transfusion is an essential tool in equine critical care and surgical practice. Indications for transfusion include severe anemia from surgical blood loss or acute hemorrhage, hemolysis due to toxins, drugs, or immune-mediated conditions, coagulopathies, and nonregenerative disorders and neonatal isoerythrolysis [6]. In horses, as in other animals, transfusions are associated with a number of inherent risks, such as transfusion reactions and the shortened survival of transfused cells. The knowledge of blood groups and alloantibodies present in an individual is therefore essential for safe transfusion of blood products. Pre-transfusion screening and blood typing are indicated to minimize the risk of incompatible RBC transfusions and prevent the immunization of the recipient against incompatible RBC antigens. In horses, blood type and cross-match compatibility should ideally always be performed to determine donor–recipient compatibility [7]. In-house hemolytic and agglutination techniques are mostly used to assay blood group factors, but, because of the lack of commercially available kits, only specialized testing laboratories can perform blood typing [2]. Cross matching methods are available as bench-top laboratory assays [8], but most methods are time-consuming, making this testing methodology impractical in an emergency situation. [3,7]. As in horses, a complete match is impossible, donors that are blood type Aa, Qa and Ca−, are preferred since they reduce the sensitization of brood mares against these RBCs antigens [3,9]. The knowledge of characteristic distribution pattern of blood groups of each breed is very useful in the effective management of blood banks and safe blood transfusion services. Data on the prevalence of blood types in an equine population and in selected breeds could aid the appropriate selection of donors and recipients. A reliable point-of-care equine blood-typing immunochromatographic method has been developed [8], but there have been few epidemiological investigations of equine blood types using monoclonal antibodies. Therefore, the aims of this study were to: (I) estimate the prevalence of Ca blood type in horses from northern Italy using the new monoclonal immunochromatographic method, (II) correlate blood types with sex and breeds of horse and (III) estimate the prevalence of anti Ca alloantibodies in Ca− horses. 

## 2. Materials and Methods 

### 2.1. Blood Samples

Blood samples collected from healthy horses housed at 3 different equestrian centers located in the province of Milan (Italy) during 2018 and 2019, were used in this study. Each equestrian center made 64, 38 and 8 privately owned horses, respectively, available for blood sample. Horses originating from several European countries. A total of 2 veterinarians and 80 owners were involved in the study. Horses were blood sampled during routine medical screening and, with owner consent, surplus whole blood samples in ethylene-diamine tetra acetic acid (EDTA) (Nuova Aptaca s.r.l, Italy) and serum samples in empty tubes (Sistema BD Vacutainer®) were used for the study. Based on the University of Milan’s animal use regulations, formal ethical approval was not needed as horses were sampled with the informed consent of the owners during routine visits for health checks. Data on sex, age and breed were collected for each horse sampled. 

### 2.2. Blood Typing

Blood types were assessed on fresh blood or on 4–6 °C stored blood within 48 h of blood collection. Ca blood type was determined with an immunochromatographic method using monoclonal antibody (Lab Test Ca, Alvedia, France) following the manufacturer’s guidelines. Briefly, 3 drops of diluent were placed into a well of a 96-well plate. Then, 10 μL of EDTA blood was added and mixed with the diluent for 15 s. The tip of an immunochromatographic strip impregnated with a Ca and control monoclonal antibody at different positions was placed into the well for 2 min, permitting the RBC suspension to diffuse to the top of the strip. The resultant line at the Ca position on the strip was graded on a scale from 0 to 4+ (0 being negative, 1 being very barely perceptible, 2 being barely perceptible, 3 clearly visible but paler than control and 4+ being equal to or stronger than the control band). A test was considered valid when a red band appeared at the control site (C) [8]. To establish the intra-assay performance of immunochromatographic strip equine lab test for Ca blood type, five blood samples (three Ca+ and two Ca−) were tested 10 times on the same day, in the same laboratory. To establish the effect of storage, 4 samples, two Ca+ and two Ca−, were tested at 24, 48 h and 7 days stored at room temperature, and 24, 48 h and 7, 14, 21, 30 days stored at 4 ± 2 °C, after collection. All results were checked by two different operators. Blood typing and alloantibodies analyses were performed at the Veterinary Research Transfusion Laboratory (REVLab), University of Milan, Italy. 

### 2.3. Alloantibody Study (Presence, Specificity and Titer)

The presence of naturally occurring anti-Ca antibodies in Ca− plasma samples was investigated using the agglutination on gel technique as previously described [8,10]. Briefly, 1% RBC-LISS (Low ionic-strength solution ID-Diluent 2 (modified LISS solution), DiaMed GmbH, Crassier FR, Switzerland) suspension from a Ca+ and Ca− blood sample was prepared. Then, 50 µL of this 1 % RBC-LISS suspension from Ca+ blood samples and 25 µL of plasma from each Ca− sample were mixed in the reaction chamber placed at the top of the polypropylene gel columns (ID-Card “NaCl enzyme test and cold agglutinins”, DiaMed Switzerland) and incubated at 37 °C for 15 min. Gel columns were centrifuged in the special column gel card centrifuge (ID-Centrifuge 24S, DiaMed Switzerland) at 80× *g* for 10 min and visually examined for agglutination. For each gel card containing 6 gel columns, an auto-control (recipient cells- recipient serum) was performed. The presence of alloantibodies against RBCs of a different blood type was identified if a macroscopic agglutination was present. The cards were visually interpreted and scored on a 5-point scale as follows: 0 = negative (no agglutination; all RBCs passed through the gel and formed a compact pellet at the bottom of the column), 1+ = most RBCs located at the bottom of the column and very few RBC agglutinates dispersed in the lower part of the gel with, 2+ = most RBCs agglutinated and dispersed in the lower half of the gel column or are dispersed throughout the gel, 3+ = RBC agglutinates dispersed in the upper portion of the gel with same RBCs forming a red line on the surface of the gel, and 4+ = all RBCs form a red line at the top of the gel [1]. Results were considered negative for samples with no agglutination and positive for samples with agglutination scores ≥1+. Samples were only considered valid if the auto-control column yielded negative results. In human transfusion medicine, the specificity or identity of an anti-RBC antibody can be determined by testing a recipient’s serum or plasma with a panel of RBC suspensions with a known antigenic composition. As a rule, serum or plasma that reacts with a RBC suspension that is positive for a given antigen, but not with a RBC suspension that is negative for that antigen, is suspected to contain antibodies against the given antigen [11]. Following this rule, samples showing agglutination when cross matched against RBCs suspension from three Ca+ horses were identified as samples with suspected natural anti-Ca antibodies. Positive samples were then cross-matched using the same technique against RBCs suspension from three Ca− horses. Samples that showed agglutination against Ca+ samples but not with Ca− samples were identified as samples containing anti–Ca antibodies [12]. 

The agglutinin titer of antibodies is defined as the highest dilution of plasma in which agglutination against Ca+ RBCs can still be detected. This was determined by creating 2-fold serial dilutions (starting from 1:2) of the plasma sample in phosphate buffered saline solution up to the highest dilution at which agglutination could be detected [13]. The gel column agglutination on gel technique was then repeated using these serodilutions. The various suspensions were incubated at 37 °C for 15 min centrifuged in the special column gel card centrifuge at 80× *g* for 10 min and evaluated for the presence and strength of agglutination as described above. The specificity of antibodies (i.e., IgG vs. IgM) was measured by treating the plasma samples with an equal volume of 0.1 M 2-mercaptoethanol and incubating at 37 °C for 60 min. 2-Mercaptoethanol abolishes the agglutination and complement-binding activities of IgM antibodies (by cleaving their disulfide bonds), allowing IgG antibodies to be detected. After incubation, the agglutinin specificity was determined based on the presence or absence of agglutination as described above [14]. 

### 2.4. Statistical Methods

The prevalence of Ca blood type and alloantibodies was calculated as the proportion of samples testing positive divided by the total number of tested samples and the proportion of sample testing Ca+ was reported as a percentage with a 95% confidence interval (95%CI). Summary statistics were performed for continuous data using Kolmogorov–Smirnov test to test for distribution and showed as mean ± standard deviation (SD) for normally distributed data. Prevalence, in Ca+ blood type between breeds represented by more than 10 subjects, were compared using chi square test for two proportions. A *p* value < 0.05 was considered significant. All statistical analyses were performed using a statistical software package (MedCalc software, version 19.1.3, Mariakerke, Belgium). 

## 3. Results

### 3.1. Population

A total of 110 whole blood samples were collected, one for each horse. Data on the signalment of the horse population are reported in Table 1. Breeds, in this horse population were 44/110 (40%) Italian Saddle horse, 12/110 (10.9%) Dutch Warmblood, 9/110 (8.2%) Hannover, 6/110 (5.5%) Selle Francais, 6/110 (5.5%) Belgian Warmblood and 6/110 (5.5%) Italian Trotter, 5/110 (4.5%) Holsteiner, 5/110 (4.5%) Westfalen, 5/110 (4.5%) Polish Warmblood, 3 (2.7%) Arabian, 2 (1.8%) Irish Sport Horses, 2 (1.8%) Andalusian Horse and 1 (0.9%) Lusitanian, 1 Oldemburg,1 Hungarian Warmblood, 1 Swedish Warmblood, and 1 Wielkopolsky. 

### 3.2. Blood Type

The prevalence of Ca+ blood types is reported in Table 2. There was no significant relationship between the Ca blood type and sex as 79.5% and 78.7% of females and males tested Ca+, respectively (*p* = 0.9239). 

There was no statistically significant difference in prevalence of Ca blood types between the two horse breeds represented by more than 10 subjects (Table 2). 

**Figure 1:**
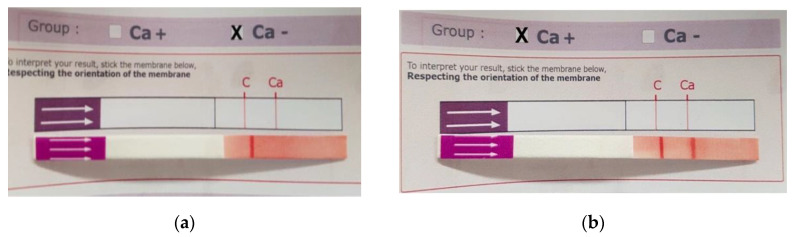
Equine Ca blood typing by immunochromatographic method. Sample Ca− (**a**) and Ca+ (**b**).

The immunochromatographic strip method was able to correctly blood type all samples. The control red band was always visible. The resultant line at the Ca position on the strip was always detectable with grades from +3 to +4 compared to color intensity of control band (Figure 1). The intra-assay performance of the immunochromatographic test was excellent, as the identical, correct blood type was recorded in all 5 samples (three Ca+ and two Ca−) tested 10 times on the same day. 

The immunochromatographic strip method was able to correctly blood-type samples stored at room temperature and at 4 ± 2 °C for up to one week and for up to 4 weeks, respectively.

### 3.3. Alloantibodies 

**Figure 2:**
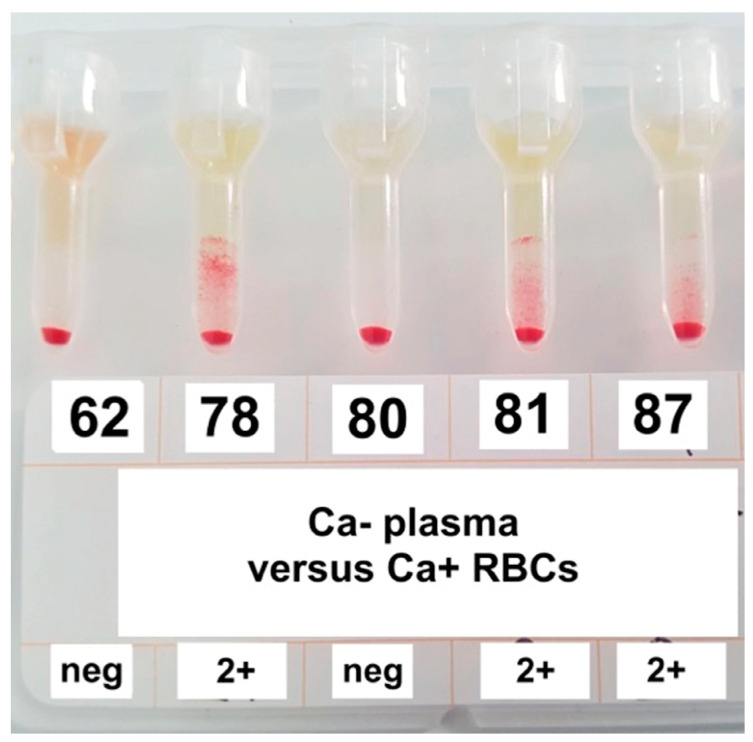
Agglutination on gel technique for anti-Ca alloantibodies detection. Gel columns show negative reaction (samples n. 62, 80) and positive reactions with strength of agglutination 2+ (samples n. 78, 81, 87).

All 23/110 samples blood typed Ca− were tested for alloantibodies. Eight of the 23 Ca− samples had detectable alloantibodies against type-Ca+ RBCs with 2 + strength of agglutination in gel column (Figure 2). In all samples, the agglutination titer was <1:2. One of the eight samples also showed a 2+ positive agglutination in gel column against RBC suspensions from three Ca− horses. Therefore, this sample was not classified as containing anti–Ca antibodies and the total number of samples with detectable anti-Ca alloantibodies was 7/23 (30.4%, 95% CI 15.6%–50.8%). The seven horses where the alloantibodies were detected were: four Italian Saddle horses, two gelding and two female, 16, 19, 20 and 16 years hold respectively; one gelding Dutch Warmblood 17 years old; one gelding Polish Warmblood 9 years old; and one gelding Hannover 18 years old.

**Figure 3:**
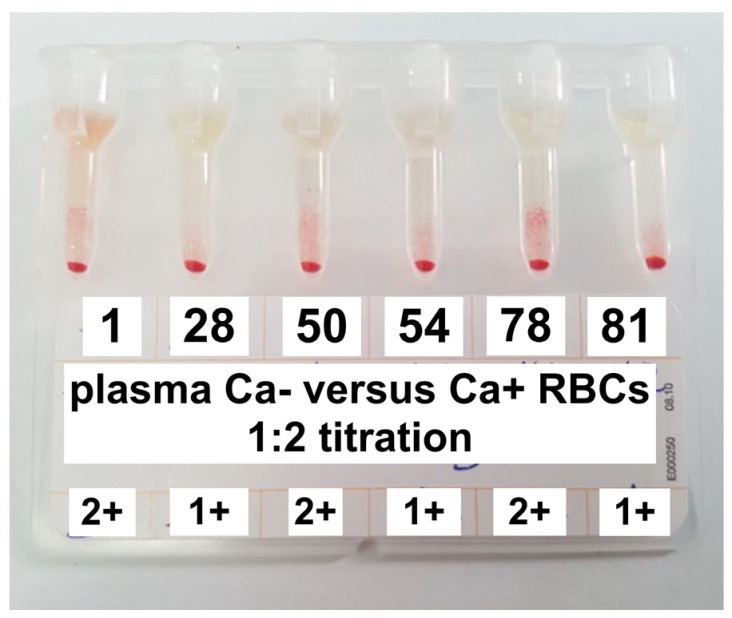
Agglutination on gel technique for anti-Ca alloantibodies titration. Gel columns show strength of agglutination 1+ (samples n. 28, 54 and 81) and 2+ (samples n. 1, 50, 78).

The seven samples were treated with 2-mercaptoethanol, and all seven samples were found to contain only IgM at an agglutination titer of 1:2 and with a strength of agglutination of +1 and +2 (Figure 3). 

## 4. Discussion

Blood typing and antibody screening prior to blood transfusion minimizes the risks of transfusion reactions and prevents immunization of recipients against incompatible RBC antigens. The distribution of blood groups varies worldwide in different regions and among different breeds. Therefore, information on the prevalence of blood types in different breeds may help in the selection of more reliable blood donors both in blood bank management and in clinical practice. There are few recent studies regarding blood type prevalence in horses. Our study evaluates the prevalence of Ca blood type and alloantibodies in horses from a region in northern Italy. This study found that of 110 blood typed horses, 79.1% were typed Ca+. This result is in concordance with previous studies that reported a similar Ca+ prevalence [15,16], but is different from the result of a recent, limited, survey on 38 horses that showed a higher (92.2%) Ca+ blood typed prevalence [8] and from the result obtained by Fenn et al. (2020) of 95.2% of Ca+ in a small group of 21 horses.

Currently, the preferred equine blood donor is Aa, Qa and Ca− because this kind of donor may decrease the risk of acute transfusion reactions and the risk of sensitizing brood mares against major RBC antigens associated with neonatal isoerythrolysis [3,8]. In horses, blood groups vary with breed, and horses of the same breed are more likely to have similar blood types. In some situations where testing is not possible, such as in a rural practice, the knowledge of the breed blood type frequencies may help in selection of the best donor candidate. In thoroughbreds, a high prevalence of Aa (98%), Ca (83%) and Qa (85%) has been reported, while Arabian breeds have high prevalence of antigens Aa (97%) and Ca (97%) but not of Qa (37%). On the other hand, standardbreds lack the Qa antigen while Morgans have a very low prevalence of Qa and Ka [16,17]. It is assumed that breeds which have undergone less vigorous breeding selection, that display a diverse array of polymorphisms and that have been bred for multiple traits, tend to have more heterogenicity in their blood types [17]. A previous study showed that 53% of Shetland ponies have the A blood type and that 52% have the Q blood type [15,18]. Horse populations in our study comprised a variety of breeds and only two breeds—Italian Saddle horse and Dutch warmblood—were represented by more than 10 subjects. In these two breeds, the Ca+ blood type prevalence was 77.3% and 58.3% respectively, with no statistically significant difference. Further investigation is required to establish the prevalence of breed-specific blood type variation. Our study highlights no significant association between blood type and sex as reported in other domestic animals such as dogs and cats [19,20].

The present study reports the prevalence of the Ca blood type, evaluated using an immunochromatographic method. In a previous study, immunochromatographic strip methods were proven to be sensitive and specific in determining Ca blood type [8]. The results of our study confirm that equine Ca+ RBCs can be identified using this rapid test. The test is easy to interpret, does not require sophisticated equipment and is suitable for use in a clinical setting. The availability of a reliable rapid diagnostic test will greatly increase the diagnostic abilities of veterinarians in the field and in emergency situations. 

In horses, red cells antigens Ca, Aa and Qa are also clinically important for their role in transfusion reactions and neonatal isoerythrolysis [3,9]. Aa and Ca antibodies are either agglutinative or hemolytic, while Qa antibody is only hemolytic [1]. The development of Ca alloantibodies after pregnancy or transfusion are less associated with the risk of acute transfusion reactions or risk of sensitizing brood mares, than Aa and Qa alloantibodies which are responsible for 90% of cases [3,5]. It has also been reported that anti-Ca alloantibodies may be protective against neonatal isoerythrolysis. In fact, it seems that Aa− mares that already have anti-Ca alloantibodies, are less like to produce antibodies against Aa antigens on the foal’s RBC [9]. A prevalence of 20% and 10% anti-RBC antibodies in Standardbred (STBD) and Thoroughbred (TB) mares, respectively, has been previously reported, with anti-Ca alloantibodies most represented [21]. In our study, the prevalence of anti-Ca alloantibodies evaluated in the 23 Ca− samples was 30.4%. This result agrees with a recent study that reported that the majority of predicted cross-matching incompatibilities were because of alloantibodies against the Aa blood type, followed by the Ca blood type [8]. A prevalence of 20% and 10% of anti-Ca alloantibodies was reported in STBD and TB mares respectively, without known exposure to Ca+ RBCs suggesting that a common environmental antigen may stimulate the production of anti-Ca antibodies [21]. Furthermore, it is reported that horses lacking Aa and Ca RBC antigens can develop antibodies against these antigens without having been sensitized [3,22].

Only low titers (1:2) of anti–Ca antibodies were found in our study, and all with weak agglutination reactions (only strength of agglutination +1 and +2 were detected). The clinical importance of horse serum alloantibodies has not been clearly established. For example, as previously mentioned, anti-Ca antibodies do not always produce adverse clinical effects [21]. Nevertheless, the risk of premature destruction of transfused RBC due to incompatible Ca alloantibodies is real [3]. Low levels of alloantibodies may be insufficient to cause significant intravascular or extravascular hemolysis, but the transfused red cells may be removed from circulation after a few days [22]. Two studies evaluating RBC lifespan after allogeneic transfusion of crossmatch-compatible blood confirmed that RBC survival time was shorter in horses than in other species. This decreased RBC lifespan may be associated with previously unidentified naturally occurring alloantibodies, perhaps with a titer too low to be detected in vitro [7,23]. The prior transfusion history of the seven Ca− horses showing antibodies in this study was unknown and also it was not known if any horse had been diagnosed with neonatal isoerythrolysis as a foal or had produced foals that developed neonatal isoerythrolysis; therefore, it is not possible to speculate about acquired antibodies. Nevertheless, IgM antibodies detection is often referred to as naturally occurring [21]. In our study, anti–Ca antibodies were all IgM, since all seven positive samples were negative after treatment with 2-mercaptoethanol. IgM antibodies activate the classical complement and the quantity of IgG or IgM and/or complement bound to the red cell, and the presence of target antigen in tissues and/or body fluids may be other factors that can influence the pathological effects of antibodies [22]. 

There are a number of limitations to this study. Firstly, is not an extensive survey but a preliminary study. In addition, the population was made up of subjects of many different breeds. In this not-homogeneous population, there were only a few subjects for each different breed, meaning that sufficient numbers were only present in two breeds for the extrapolation of data about the breed prevalence of the Ca group. However, data regarding the Italian Saddle Horse and Dutch Warmblood have also been provided. Furthermore, only agglutinating antibodies were evaluated and not hemolytic ones. Nevertheless, it should be noted that during cross-matching evaluation, all horses that showed anti-Ca agglutinins also had hemolytic anti-Ca antibodies [1].

## 5. Conclusions

The knowledge of blood group and the presence of alloantibodies are crucial to make transfusion medicine safer in horses. Due to the high number of antigens present on the horse RBC, assessment of blood compatibility is very challenging and is mainly based on cross-matching. Knowledge of the prevalence of blood groups in the various breeds helps in the selection of the most suitable donor. This study brings new information about the prevalence of the Ca blood group in both the Italian Saddle Horse and Dutch Warmblood. In emergency conditions and in certain clinical situations where testing blood compatibility is not possible, such as in a rural practice, the knowledge of the breed blood type frequencies may help selecting the best donor candidate. It also reports new data regarding the titration of alloantibodies in type Ca− subjects (30.4%).

Additional studies, including larger numbers of subjects, are needed to clarify the prevalence of the Ca blood type in various horse breeds and to quantify the amount and the clinical significance of alloantibodies in this species. 

## Figures and Tables

**Table 1:** Demographic data of 110 horses blood typed using an immunochromatographic method.

Study Population	Number (%)	Age (Years), Mean ± SD	Stallion	Female	Gelding
**Horses**	110/110 (100%)	13.8 ± 5.3	6 (5.5%)	44 (40%)	60 (54.5%)

SD = standard deviation.

**Table 2:** Ca+ blood type prevalence (95% confidence interval) in the whole horse study population, in Italian Saddle horse and in Dutch Warmblood breeds.

Horse	Type Ca+	*p*-Value
Whole horse population (*n* = 110)	87 (79.1%) (95% CI 70.5%–85.6%)	
Italian Saddle Horse (n = 44)	34 (77.3%) (95% CI 63.0%–87.1%)	0.1916 *(ISH vs DW)*
Dutch Warmblood (n = 12)	7 (58.3%) (95% CI 31.9%–80.6%)	

N = number; CI = confidence interval. Statistically significant *p* value < 0.05.
